# Sexual selection in mushroom-forming basidiomycetes

**DOI:** 10.1098/rspb.2010.1110

**Published:** 2010-07-14

**Authors:** Bart P. S. Nieuwenhuis, Alfons J. M. Debets, Duur K. Aanen

**Affiliations:** Droevendaalsesteeg 1, 6708 PB, Wageningen, The Netherlands

**Keywords:** sexual selection, basidiomycota, female choice, male–male competition, dikaryon, Buller phenomenon

## Abstract

We expect that sexual selection may play an important role in the evolution of mushroom-forming basidiomycete fungi. Although these fungi do not have separate *sexes*, they do play female and male *roles*: the acceptance and the donation of a nucleus, respectively. The primary mycelium (monokaryon) of basidiomycete fungi, growing from a germinating sexual spore, is hermaphroditic, but it loses female function upon the acceptance of a second nucleus. The resulting dikaryon with two different nuclei in each cell retains a male potential as both nuclei can fertilize receptive mycelia. We tested the occurrence of sexual selection in the model species of mushroom-forming basidiomycetes, *Schizophyllum commune*, by pairing monokaryons with fully compatible dikaryons. In most pairings, we found a strong bias for one of the two nuclei although both were compatible with the monokaryon when paired alone. This shows that sexual selection can occur in mushroom-forming basidiomycetes. Since the winning nucleus of a dikaryon occasionally varied depending on the receiving monokaryon, we infer that sexual selection can operate through choosiness of the receiving individual (analogous to female choice). However, in other cases the same nucleus won, irrespective of the receiving monokaryon, suggesting that competition between the two nuclei of the donating mycelium (analogous to male–male competition) might also play a role.

## 1. Introduction

Sexual selection is defined as the component of natural selection associated with variation in reproductive success caused by competition for access to gametes of the opposite sex [1,2]. It is reflected in competition between individuals of the same sex for mating (usually strongest in males: ‘male–male competition’) and preference for some individuals as mates (usually strongest in females: ‘female choice’). Sexual selection is known to be of importance in the animal and plant kingdom [3–5], but so far this has not been recognized in fungi (but see [6]). In plants and animals the traits and behaviours associated with sexual selection are often quite elaborate, but in fungi such traits are more difficult to observe. In this paper, we show that sexual selection occurs in the basidiomycete fungus *Schizophyllum commune*.

**Figure 1.**
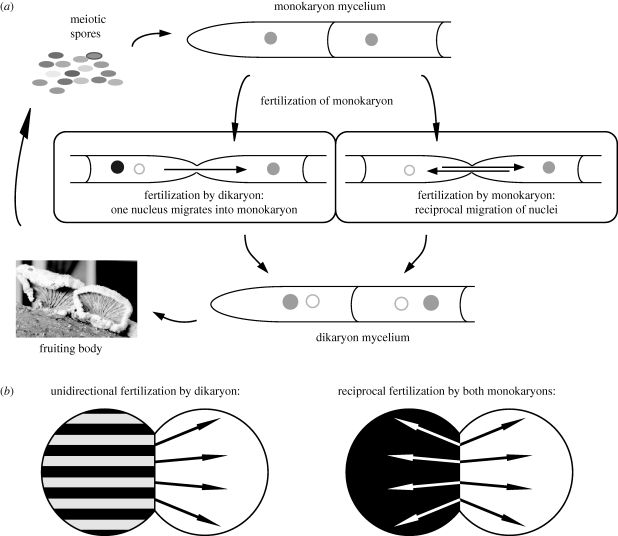
Life cycle and fertilization of *Schizophyllum commune*. Representation of life cycle of *S. commune* with a monokaryon–monokaryon mating and dikaryon–monokaryon mating at (*a*) the hyphal level and (*b*) the mycelium level.

The life cycle of most basidiomycetes encompasses two distinct phases: those of the monokaryon and the dikaryon. Initially, a meiotic haploid spore germinates, giving rise to a mycelium with uninucleate cells, the monokaryon. This mycelium can grow vegetatively and, when it meets another monokaryon of the same species, hyphal fusions occur between the two mycelia (figure 1). At that moment fertilization of the mycelium can occur. In most mushroom-forming basidiomycetes, fusion is followed by exchange of nuclei but not cytoplasm [7,8], resulting in a mycelium with binucleate cells, the dikaryon. Nuclei migrate from the contact zone through the whole receiving mycelium [9]. The exact process of dikaryotization is unknown, but it must involve many nucleus duplications because the outcome of dikaryotization is that all cells of both receiving mycelia contain both nucleus types (figure 1*b*). Just like the monokaryon, the dikaryon can grow vegetatively, but it is also able to form sexual fruiting bodies (the mushrooms). In the fruiting bodies, the two nuclei fuse, directly after which meiotic spores are produced. A dikaryon can no longer accept other nuclei, but it can still donate nuclei to a monokaryon [10,11], a phenomenon called the ‘Buller phenomenon’.

Even though basidiomycetous fungi are considered to have no sexes [12,13], clear male and female roles can be distinguished in their general life cycle [14,15]. Using the common criterion that male and female gametes are defined by small and large size, respectively [16], the acceptance of a nucleus by a large mycelium that contributes all cytoplasm can be seen as a female-like function, and the donation of a nucleus as a male-like function. Previously, people have referred to mating types in basidiomycetous fungi as being different sexes (e.g. [17]). Note that we do not. We will treat mating types as sexual compatibility systems, comparable to self-incompatibility systems in plants. We will go into more detail on this topic in §4. The male- and female-like functions imply that a monokaryon is hermaphroditic, but that it can function only once as a female during mating, while after having been fertilized it retains its male potential via the Buller phenomenon. Furthermore, spores that have not germinated can also act as males by fertilizing a monokaryotic mycelium [18]. According to this view, the nucleus functions as the male gamete and the receiving mycelium as the female gamete. The consequence of this is that in nature the ratio of male and female functions is strongly male-biased [19].

Sexual selection is expected to occur during a dikaryon–monokaryon (di–mon) mating because both nuclei (analogous to male gametes) of the dikaryon are able to fertilize the receiving monokaryon (analogous to the female gamete). An important prediction is that the monokaryon should be choosy as a female: after fertilization by a nucleus, it is engaged in a lifelong relationship with that nucleus. In other words, the monokaryon can play its female role only once. In contrast, the nuclei of the dikaryon are expected to be promiscuous as the fertilization of a monokaryon is essentially cost-free and they can play the male role over and over again. Therefore, the two nuclei compete for fertilization, which potentially selects for traits that increase success in male–male competition. It has been shown that systematic differences in mating success between the two nuclei of a dikaryon can occur in di–mon matings [20–22]. However, this has not been recognized as sexual selection and has not been studied systematically for many strains. Furthermore, it is unknown whether this difference is based on female choice or male–male competition.

Here, we test the occurrence of sexual selection in *S. commune*. To show its occurrence, we investigate if selection during matings occurs based on a genetic characteristic that favours one nucleus type over another in fertilization. Assuming that sexual selection occurs, we expect to observe consistent differences between nuclei in their mating success in a given pairing. Because fungi can be multiplied clonally, we have been able to perform the exact same mating in many replicates. Furthermore, owing to the hermaphroditic character of the nuclei, we can use the male and female characteristics of the same genotype to experimentally distinguish between the two main causes of sexual selection: male–male competition and female choice. With male–male competition one of the two nuclei in the dikaryon should have a consistently higher fertilization success, irrespective of the receiving monokaryon. In contrast, with female choice, which nucleus wins will depend on the receiving monokaryon.

## 2. Material and methods

### (a) Strains, media and growth conditions

In this research, six different monokaryotic strains were used, designated A through F, which were derived as follows. Six dikaryotic mycelia were isolated from fresh fruiting bodies of *S. commune*, collected in the Netherlands (A, B, E and F), Germany (C) and Slovenia (D), and were fruited in the laboratory [23]. From each fruiting body, we isolated a monokaryon originating from a single spore. To exclude effects of cytoplasmic elements, we placed each nucleus in the same cytoplasmic background. For this, we crossed each of the six monokaryons in their male function with a seventh monokaryon to establish a dikaryon. We de-dikaryotized these dikaryons using protoplast regeneration according to the method of de Vries & Wessels [24]. During this process monokaryotic mycelia can be obtained that only possess one of the nucleus types of the dikaryon. From the retrieved monokaryons we selected the original monokaryons based on the mating types.

Furthermore, for each strain we created a transformant that contains a dominant resistance marker to the antibiotic nourseothricin (construct pGEMNour; kindly provided by Luis Lugones) using protocols described in van Peer *et al.* [25]. All strains were grown at 27°C in the dark on minimal medium [26].

### (b) Dikaryon–monokaryon matings

We created all 15 possible dikaryons from the six monokaryon combinations [27]. To control for marker effects and role in dikaryon formation, for each pair of monokaryons four types were created: with either nucleus containing the resistance marker and with either nucleus as receiving mycelium (e.g. A_res_B, AB_res_, BA_res_ and B_res_A; the first letter indicates the receiving mycelium and the second the donating mycelium). All dikaryons were tested against the four monokaryons with which no nucleus was shared (see table 1), with 10 replicates per combination. In total 2400 pairings were performed (15 dikaryons × 4 treatments × 4 receiving monokaryons × 10 replicas). The actual crosses were performed by placing a plug of the dikaryon 5 mm from the edge of a 3-day-old monokaryon. After 5 days of incubation, two mycelium plugs from the initially monokaryotic mycelium—which by then had been dikaryotized completely—were taken and tested for nourseothricin resistance. Because the marker is dominant, the dikaryon can be directly tested for growth on plates containing nourseothricin (15 µg ml^−1^). For a subset also, mating type was used as a marker [28] to confirm that the marker functioned correctly. No incongruence was found between the resistance marker and mating types.

**Table 1.** Results for all dikaryon–monokaryon matings. The fertilizing dikaryon is given in the rows and the receiving monokaryon in the columns. Each intersection shows the nucleus that performed most of the fertilizations (*p* < 0.0009; *n* = 40). n*.*s*.* indicates there was no significant deviation from 1 : 1 ratio. The ratio of the winning nucleus is also given. When there was no significant difference, the ratio of the first nucleus mentioned is given. The intersections indicated with ‘—’ were not tested because one of the nuclei was shared between dikaryon and monokaryon.

	A	B	C	D	E	F
AB	—	—	A 1.00	B 0.85	— ^a^	—^a^
AC	—	C 0.90	—	n.s. 0.70	C 0.83	n.s. 0.30
AD	—	D 0.90	D 0.90	—	D 0.90	n.s. 0.55
AE	—	E 0.85	A 1.00	A 1.00	—	n.s. 0.30
AF	—	F 1.00	F 0.90	A 0.80	A 0.80	—
BC	B 0.80	—	—	n.s. 0.65	B 0.80	n.s. 0.25
BD	n.s. 0.37	—	D 1.00	—	D 0.98	D 1.00
BE	B 1.00	—	B 0.98	E 0.90	—	E 0.80
BF	B 0.80	—	F 0.80	F 1.00	F 1.00	—
CD	D 0.85	D 0.85	—	—	D 0.95	n.s. 0.60
CE	n.s. 0.60	C 0.85	—	C 0.93	—	C 0.95
CF	n.s. 0.70	n.s. 0.70	—	F 0.93	F 0.88	—
DE	D 0.83	D 0.85	D 1.00	—	—	D 0.88
DF	F 0.75	F 0.93	n.s. 0.53	—	D 0.90	—
EF	F 0.85	F 0.95	F 1.00	F 0.88	—	—

^a^Owing to contaminations of the samples no data for these crosses were obtained.

## 3. Results

We performed all possible dikaryon–monokaryon matings between six monokaryon strains and all their 15 dikaryon combinations (table 1). For each mating we established the frequency of fertilization per nucleus type. We did not find an effect for marker (e.g. A_res_B or AB_res_) nor for maternal effects (i.e. whether a nucleus in the fertilizing dikaryon descended from the receiving or from the donating monokaryon—e.g. AB or BA) upon the fertilizing success of nuclei. Therefore, we treated all four kinds of dikaryon containing the same nuclei as additional replicates. We first give the results of each mating individually and will then subsequently discuss the results from the dikaryon (male) point of view and from the receiving monokaryon (female) point of view.

For 46 out of 58 di–mon matings, we found a ratio that significantly differed from 1 : 1, after Bonferroni correction for multiple (*n* = 58) replicates (binomial test, *p* < 0.0009, *n* = 40), which indicates that selection of one of the two nuclei occurred. Across all pairings, the mean value of the most successful nucleus was 0.85 (s.d. 0.124).

For six of the 15 tested dikaryotic strains, the nucleus fertilizing ‘male’ depended on the receiving mycelium (female). For nine dikaryons the same nucleus was always most successful, with all four receiving monokaryons. To test whether this result was caused by an inherent difference between the two nuclei irrespective of receiving monokaryon, or by the low number of tested receiving monokaryons (four), we tested four of these strains (BD, CE, DE and EF) with five additional receiving monokaryons (strains G–K, each originating from a different dikaryon; G collected in Brazil and H–K in the Netherlands). For one dikaryon (CE) in one pairing this time it was the other nucleus that was more successful, whereas for the other dikaryons again the same nucleus always won (data not shown).

From the receiving monokaryon perspective, half of the monokaryons (B, C and D) showed a clear transitive hierarchy in fertilizing nuclei (if nucleus Y was preferred over X, and Z over Y, then Z was also preferred over X). A comparison of the ranking between these three strains showed no clear pattern that would indicate a shared preference (rankings given in table 2). Monokaryon F had too few comparisons to make a complete ranking. For the receiving monokaryons A and D preference was not hierarchical.

**Table 2.** Fertilization ranking per receiving monokaryon. For each receiving monokaryon, a ranking is indicated of the success of fertilizing nuclei in Buller pairings. For four receiving monokaryons a ranking is found; for A and E no ranking can be made (see also table 1). The ranking for monokaryon F is based on few comparisons owing to many non-significant interactions.

A	no ranking								
B	F	>	D	>	C	>	E	>	A
C	F	=	D	>	A	>	B	>	E
D	A	>	F	>	C	>	E	>	B
E	no ranking								
F	D	>	C	>	E	>	B		

## 4. Discussion

Sexual selection acts in mushrooms. Our results show that a highly reproducible strong bias for either one of the two potentially fertilizing nuclei in natural isolates of *S. commune* exists—indicating sexual selection—and that this bias depends partly on the receiving mycelium—indicating female choice. Next to female-dependent fertilization, for nine dikaryons we found that always the same nucleus performed the fertilization, irrespective of the female. This indicates that some nuclei are more successful males than others, either in being chosen, or in direct competition with other nuclei.

The separation of sexual selection in male–male competition and female choice is somewhat artificial and both processes are not mutually exclusive. Only when one of the two sexes is in full control of the fertilization will such a distinction be applicable. Our results show that in some di–mon matings female choice acts, because the nucleus in the dikaryon chosen depends on the receiving monokaryon. Even though female choice can be shown with our experiment, unfortunately, we cannot be so conclusive about male–male competition. When the same nucleus in a dikaryon is always more successful, irrespective of the receiving monokaryon, this might be caused by a direct interaction between the two nuclei (i.e. male–male competition). However, it is still possible that female choice acts, but that all receiving mycelia have the same preference. These two processes cannot be distinguished here.

It is unclear on which criteria the observed selection, be it driven by female choice or by male–male competition, is based. If selection would be based on a single quantitative trait, then we should be able to create a hierarchy; if Y is preferred over X, and Z over Y, then Z should be preferred over X. The same goes for competition. For half of the receiving monokaryons a hierarchy cannot be made (table 2). This either means that competition and preference act at the same time in opposite directions, or that preference depends on a nonlinear trait or multiple traits. An example of the latter might be that next to a hierarchical trait heterozygosity also is selected for. A candidate trait might be the mating type. For the Buller phenomenon the mating type locus (or loci) has been suggested as a trait for selection, in which the nucleus in the dikaryon that is more different at this locus in relation to the receiving monokaryon wins [21,29].

Basidiomycete fungi have a sexual compatibility system, comparable to the self-incompatibility system of angiosperm plants, determined by one or two mating type loci. Only when the mating type factors are different will successful mating occur and consequently will a dikaryon always be heterozygous at the mating type locus or loci. Because of the high diversity in mating type alleles in *S. commune* (like in many mushroom-forming basidiomycetes), about 97 per cent (mon–mon) and 95 per cent (di–mon) of the matings between two individuals in nature will be fully compatible [30]. Nuclear exchange and maintenance of the dikaryon phase are mediated by the interaction of the genes of the mating types of the interacting nuclei (reviewed in [31]) and can partly be used to predict nucleus selection in isogenic lines [20,21].

The B-locus, coding for one of the two mating type factors, has also been identified as an important determinant for recovery of monokaryons from dikaryons after artificial de-dikaryotization using protoplast regeneration (see §2; see also [24,32]). Raper [32] found a transitive hierarchy of recovered nuclei, which was caused by an interaction between the two nuclei in a dikaryon. It was suggested by Nogami *et al.* [33] that the recovery success of nuclei after de-dikaryotization is correlated with the relative success of nuclei in Buller pairings; this could be interpreted as an example of male–male competition. Using three strains of *Pholiota nameko*, they observed the same hierarchy for monokaryon recovery as for Buller fertilization, but because of the low number of strains used, each time only two strains could be compared, and only for one receiving monokaryon. Even though we did not find a consistent hierarchy in our matings, the described interaction between the nuclei could act during a Buller mating (table 2). This discrepancy between these studies and ours can be caused by their use of highly inbred strains that were only different for mating types, whereas we used natural isolates. It has been found that other genes than the mating type genes also affect nuclear success in Buller matings [29, p. 123]; B. P. S. Nieuwenhuis 2008 unpublished data), which might be an explanation for the non-hierarchical pattern in the Buller matings reported in this paper.

Sexual selection is considered an important component of natural selection driving evolution in many different groups of sexual organisms, but to our knowledge it has until now not been recognized in filamentous fungi. The strong preferences that we found in natural isolates show that sexual selection is potentially very significant in the life cycle of mushrooms, in which di–mon matings are likely to be frequent [29,34], and that it should be considered when studying mushrooms. Recently, Rogers & Greig [6] showed in a very elegant experiment with the single-celled fungus *Saccharomyces cerevisiae* that selection in a very sex-biased environment also leads to sexual selection. In this experiment, female preference for high pheromone levels led to the evolution of increased pheromone production. However, in this species such bias in natural situations is not very likely.

It will be interesting to study how sexual selection affects other fitness components of the resulting dikaryon. Because fertilization has direct effects on the receiving mycelium (e.g. changed growth rate: [23]; protein expression: [35]) and indirect effects through offspring fitness, fitness measurements (cf. [36]) should be performed on the dikaryon itself as well as on monokaryons originating from basidiospores from mushrooms formed by the dikaryon. To understand the evolutionary advantage of female choice and to explore whether male–male competition can arise, more needs to be known on the ecology of mushroom species. How long is the monokaryon phase? How many monokaryotic and dikaryotic individuals will a mycelium meet? What is the cost of inbreeding?

Our findings show that sexual selection is more broadly present than was previously thought and that it also acts in fungi. This example confirms that, whenever variation occurs in fertilization success between individuals, no matter how cryptic, a potential for the evolution of sexually selected traits exists. Bateman [37] suggested that selection between males and related effects may have influenced the evolution of animals and plants in various ways for which much support has been found over the years. Our findings indicate that this might also be true for fungi.
